# Crystal structures of 2-[(4,6-di­amino­pyrimidin-2-yl)sulfan­yl]-*N*-(naphthalen-1-yl)acetamide and 2-[(4,6-di­amino­pyrimidin-2-yl)sulfan­yl]-*N*-(4-fluoro­phen­yl)acetamide

**DOI:** 10.1107/S2056989017001293

**Published:** 2017-01-31

**Authors:** S. Subasri, Timiri Ajay Kumar, Barij Nayan Sinha, Venkatesan Jayaprakash, Vijayan Viswanathan, Devadasan Velmurugan

**Affiliations:** a Centre of Advanced Study in Crystallography and Biophysics, University of Madras, Guindy Campus, Chennai 600 025, India; b Department of Pharmaceutical Sciences & Technology, Birla Institute of Technology, Mesra, Ranchi 835 215, Jharkhand, India

**Keywords:** crystal structure, di­amino­pyrimidin-2-yl, thio­acetamide, hydrogen bonding, inversion dimers

## Abstract

The title compounds, C_16_H_15_N_5_OS, (I), and C_12_H_12_FN_5_OS, (II), are [(di­amino­pyrimidine)­sulfan­yl]acetamide derivatives. In (I), the pyrimidine ring is inclined to the naphthalene ring system by 55.5 (1)°, while in (II), the pyrimidine ring is inclined to the benzene ring by 58.93 (8)°. In (II), there is an intra­molecular N—H⋯N hydrogen bond and a short C—H⋯O contact. In the crystals of (I) and (II), mol­ecules are linked by pairs of N—H⋯N hydrogen bonds, forming inversion dimers with *R*_2_^2^(8) ring motifs. In the crystal of (I), the dimers are linked by bifurcated N—H⋯(O,O) and C—H⋯O hydrogen bonds, forming layers parallel to (100). In the crystal of (II), the dimers are linked by N—H⋯O hydrogen bonds, also forming layers parallel to (100). The layers are linked by C—H⋯F hydrogen bonds, forming a three-dimensional architecture.

## Abstract 2

The two title compounds are (di­amino­pyrimidin-2-yl)thio­acetamide derivatives. In the first structure, the pyrimidine ring is inclined to the naphthalene ring system by 55.5 (1)°, while in the second, the pyrimidine ring is inclined to the benzene ring by 58.93 (8)°. In the crystals of both compounds, mol­ecules are linked by pairs of N—H⋯N hydrogen bonds, forming inversion dimers with 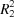
(8) ring motifs.

## Chemical context   

As a result of the innate ability of bacteria to develop resistance to available anti­biotics, there is a critical need to develop new agents to treat more strains that are resilient. Several classes of di­amino­pyrimidines have been reported as new therapeutic agents. Derivatives of di­amino­pyrimidines also exhibit anti-cancer activity (Xu *et al.*, 2010 ▸), immune suppressant activity (Blumenkopf *et al.*, 2002 ▸), hair-growth-stimulant properties, anti-bacterial (Kandeel *et al.*, 1994 ▸) and potential anti-microbial properties (Holla *et al.*, 2006 ▸). They are also used as potential anti-AIDS agents (Nogueras *et al.*, 1993 ▸) and anti-viral agents (Hocková *et al.*, 2004 ▸). In this connection, the title 4,6-di­amino­pyrimidine-based analogues have been synthesized as potential anti­viral agents against dengue for targeting NS2B/NS3 protease.

## Structural commentary   

**Figure 1:**
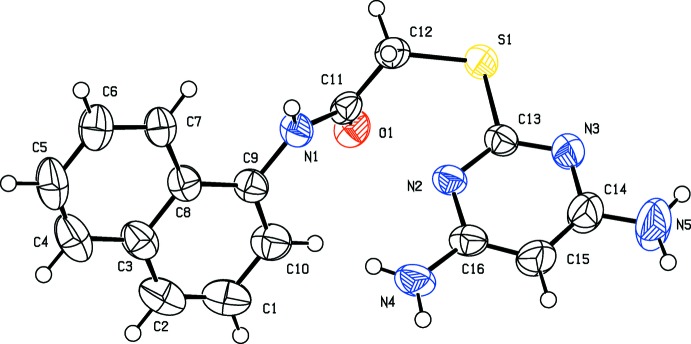
The mol­ecular structure of compound (I), showing the atom labelling and displacement ellipsoids drawn at the 50% probability level.

The mol­ecular structure of compound (I) is shown in Fig. 1 ▸. The pyrimidine ring is twisted with respect to the thio­acetamide unit with the N1—C11—C12—S1 torsion angle being 140.88 (18)°. The pyrimidine ring (C13–C16/N2/N3) makes a dihedral angle of 55.5 (1)° with the naphthalene ring system (C1–C10). The amine nitro­gen atoms, N4 and N5, deviate by 0.0235 and 0.0291 Å, respectively, from the plane of the pyrimidine ring.
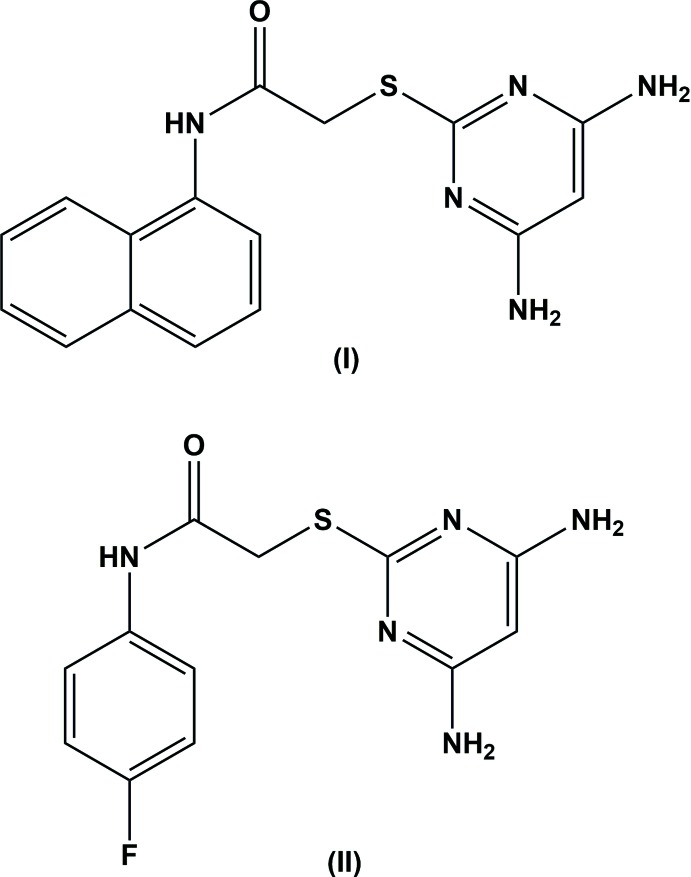


**Figure 2:**
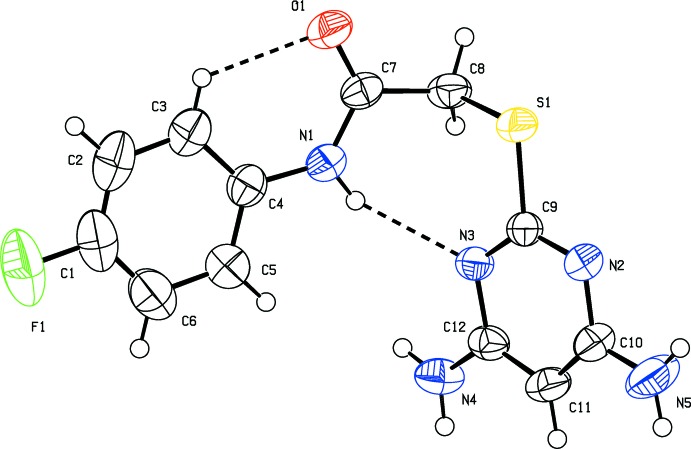
The mol­ecular structure of compound (II), showing the atom labelling and displacement ellipsoids drawn at the 50% probability level.

The mol­ecular structure of compound (II) is shown in Fig. 2 ▸. Here, the pyrimidine ring is twisted with respect to the thio­acetamide unit with the N1—C7—C8—S1 torsion angle being −82.44 (14)°. The pyrimidine ring (C9–C12/N2/N3) makes a dihedral angle of 58.93 (8)° with the benzene ring (C1–C6). The amine nitro­gen atoms, N4 and N5, deviate by 0.0247 and 0.0564 Å, respectively, from the pyrimidine ring. In compound (II), there is an intra­molecular N—H⋯N hydrogen bond and a short C—H⋯O inter­action present (Table 2 ▸ and Fig. 2 ▸).

## Supra­molecular features   

**Figure 3:**
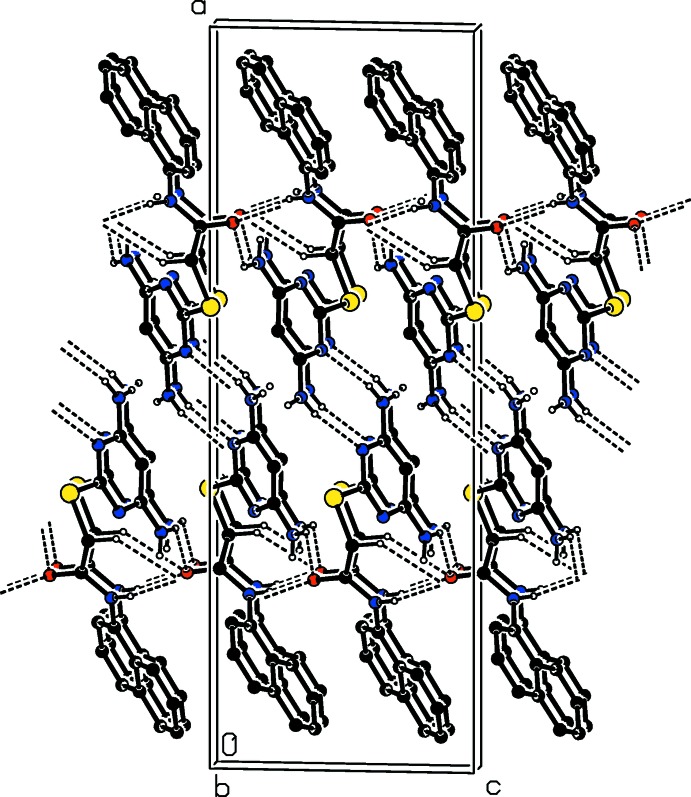
A view along the *b* axis, of the crystal packing of compound (I). Hydrogen bonds are shown as dashed lines (see Table 1 ▸). For clarity, only the NH and NH_2_ hydrogens and the C-bound H atoms involved in hydrogen bonding have been included.

In the crystal of compound (I), mol­ecules are linked by pairs of N5—H5*A*⋯N3^i^ hydrogen bonds, forming inversion dimers with an 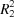
(8) ring motif (Table 1 ▸ and Fig. 3 ▸). The dimers are linked by bifurcated N—H⋯(O,O) and C—H⋯O hydrogen bonds, forming layers parallel to the *bc* plane (Table 1 ▸ and Fig. 3 ▸).

**Figure 4:**
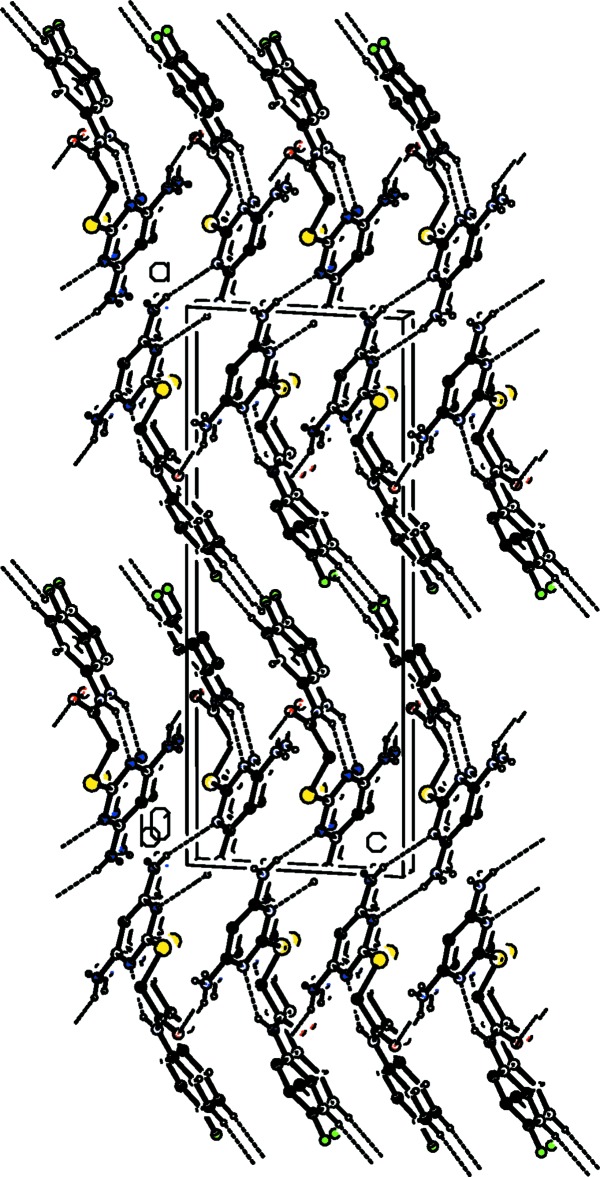
The crystal packing of compound (II) viewed along the *b* axis. Hydrogen bonds are shown as dashed lines (see Table 2 ▸). For clarity, only the NH and NH_2_ hydrogens and the C-bound H atoms involved in hydrogen bonding have been included.

In the crystal of compound (II), inversion dimers, with an 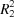
(8), ring motif, are also formed *via* pairs of N5—H5*A*⋯N2^i^ hydrogen bonds (Table 2 ▸ and Fig. 4 ▸). This time the dimers are linked by N—H⋯O hydrogen bonds and also form layers parallel to the *bc* plane (Table 2 ▸ and Fig. 4 ▸). The layers are linked by C—H⋯F hydrogen bonds, forming a three-dimensional architecture (Table 2 ▸ and Fig. 4 ▸).

## Database survey   

A search of the Cambridge Structural Database (Version 5.37, update May 2016; Groom *et al.*, 2016 ▸) for 2-(pyrimidin-2-yl)-*N*-phenyl­acetamide yielded only five hits. They include two 4,6-di­methyl­pyrimidine analogues *viz.* 2-(4,6-di­methyl­pyrimidin-2-ylsulfan­yl)-*N*-phenyl acetamide (DIWXAJ; Gao *et al.*, 2008 ▸) and *N*-(2-chloro­phen­yl)-2-(4,6-di­methyl­pyrimidin-2-ylsulfan­yl)acetamide (QOTQEW; Li *et al.*, 2009 ▸), and three 4,6-di­amino­pyrimidine compounds *viz.* 2-[(4,6-di­amino­pyrim­idin-2-yl)sulfan­yl]-*N*-2-methyl­phen­yl)acetamide (GOKWIO; Subasri *et al.*, 2014 ▸), 2-[(4,6-di­amino­pyrimidin-2-yl)sulfan­yl]-*N*-(3-nitro­phen­yl)acetamide (Subasri *et al.*, 2014 ▸) and 2-[(4,6-di­amino­pyrimidin-2-yl)sulfan­yl]-*N*-(2-chloro­phen­yl)acetamide (Subasri *et al.*, 2014 ▸).

## Synthesis and crystallization   

**Compound (I):** To a solution of 4,6-di­amino-pyrimidine-2-thiol (0.5 g, 3.52 mmol) in 25 ml of ethanol, potassium hydroxide (0.2 g, 3.52 mmol) was added and the mixture refluxed for 30 min. Then 3.52 mmol of 2-chloro-*N*-(naphthalen-1-yl)acetamide was added and the mixture refluxed for 2.5 h. On completion of the reaction (monitored by TLC), the ethanol was evaporated *in vacuo* and cold water was added. The precipitate that formed was filtered and dried to give compound (I) as a crystalline powder (yield 92%).

**Compound (II):** To a solution of 4,6-di­amino-pyrimidine-2-thiol (0.5 g, 3.52 mmol) in 25 ml of ethanol, potassium hydroxide (0.2 g, 3.52 mmol) was added and the mixture refluxed for 30 min. Then 3.52 mmol of 2-chloro-*N*-(4-fluoro­phen­yl)acetamide was added and the mixture refluxed for 4 h. On completion of the reaction (monitored by TLC), ethanol was evaporated in vacuo and cold water was added and the precipitate formed was filtered and dried to give compound (II) as a crystalline powder (yield 88%).

Colourless block-like crystals were obtained by slow evaporation of a solution in CH_3_OH for compound (I) and C_4_H_8_O_2_ for compound (II).

## Refinement   

Crystal data, data collection and structure refinement details are summarized in Table 3 ▸. For both compounds the hydrogen atoms were placed in calculated positions and refined as riding: C—H = 0.93–0.97 Å, N—H = 0.86 Å with *U*_iso_(H) = 1.2*U*_eq_(N,C).

## Supplementary Material

Crystal structure: contains datablock(s) global, I, II. DOI: 10.1107/S2056989017001293/su5347sup1.cif

Structure factors: contains datablock(s) I. DOI: 10.1107/S2056989017001293/su5347Isup2.hkl

Structure factors: contains datablock(s) II. DOI: 10.1107/S2056989017001293/su5347IIsup3.hkl

Supporting information file. DOI: 10.1107/S2056989017001293/su5347Isup4.cml

Supporting information file. DOI: 10.1107/S2056989017001293/su5347IIsup5.cml

CCDC references: 1529608, 1529607

Additional supporting information:  crystallographic information; 3D view; checkCIF report

## Figures and Tables

**Table 1:** Hydrogen-bond geometry (Å, °) for (I)

*D*—H⋯*A*	*D*—H	H⋯*A*	*D*⋯*A*	*D*—H⋯*A*
N5—H5*A*⋯N3^i^	0.86	2.27	3.110 (4)	167
N1—H1*A*⋯O1^ii^	0.86	2.05	2.890 (3)	165
N4—H4*B*⋯O1^iii^	0.86	2.36	2.964 (3)	127
C12—H12*A*⋯O1^ii^	0.97	2.58	3.408 (3)	143

Symmetry codes: (i) 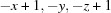; (ii) 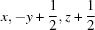; (iii) 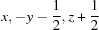.

**Table 2:** Hydrogen-bond geometry (Å, °) for (II)

*D*—H⋯*A*	*D*—H	H⋯*A*	*D*⋯*A*	*D*—H⋯*A*
N1—H1⋯N3	0.86	2.25	2.990 (2)	145
C3—H3⋯O1	0.93	2.31	2.903 (2)	121
N5—H5*A*⋯N2^i^	0.86	2.29	3.139 (2)	169
N4—H4*A*⋯O1^ii^	0.86	2.23	2.9852 (18)	146
C2—H2⋯F1^iii^	0.93	2.48	3.404 (3)	172

Symmetry codes: (i) 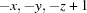; (ii) 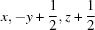; (iii) 
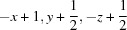
.

**Table 3:** Experimental details

	(I)	(II)
Crystal data		
Chemical formula	C_16_H_15_N_5_OS	C_12_H_12_FN_5_OS
*M*_r_	325.39	293.33
Crystal system, space group	Monoclinic, *P*2_1_/*c*	Monoclinic, *P*2_1_/*c*
Temperature (K)	293	293
*a*, *b*, *c* (Å)	25.1895 (16), 6.9411 (4), 8.9697 (6)	21.7358 (7), 7.3726 (3), 8.4487 (3)
β (°)	90.943 (4)	93.092 (1)
*V* (Å^3^)	1568.08 (17)	1351.93 (9)
*Z*	4	4
Radiation type	Mo *K*α	Mo *K*α
μ (mm^−1^)	0.22	0.25
Crystal size (mm)	0.30 × 0.25 × 0.20	0.31 × 0.25 × 0.20

Data collection		
Diffractometer	Bruker SMART APEXII area-detector	Bruker SMART APEXII area-detector
Absorption correction	Multi-scan (*SADABS*; Bruker, 2008 ▸)	Multi-scan (*SADABS*; Bruker, 2008 ▸)
*T*_min_, *T*_max_	0.752, 0.831	0.652, 0.753
No. of measured, independent and observed [*I* > 2σ(*I*)] reflections	14522, 3849, 2095	12316, 3312, 2829
*R*_int_	0.063	0.025
(sin θ/λ)_max_ (Å^−1^)	0.669	0.667

Refinement		
*R*[*F* ^2^ > 2σ(*F* ^2^)], *wR*(*F* ^2^), *S*	0.052, 0.153, 0.98	0.037, 0.109, 1.05
No. of reflections	3849	3312
No. of parameters	208	181
H-atom treatment	H-atom parameters constrained	H-atom parameters constrained
Δρ_max_, Δρ_min_ (e Å^−3^)	0.38, −0.23	0.22, −0.22

Computer programs: *APEX2* and *SAINT* (Bruker, 2008 ▸), *SHELXS97* (Sheldrick, 2008 ▸), *SHELXL2016* (Sheldrick, 2015 ▸) and *PLATON* (Spek, 2009 ▸).

## supplementary crystallographic information

### (I) 2-[(4,6-Diaminopyrimidin-2-yl)sulfanyl]-N-(naphthalen-1-yl)acetamide Crystal data

**Table:**

C_16_H_15_N_5_OS	*F*(000) = 680
*M**_r_* = 325.39	*D*_x_ = 1.378 Mg m^−^^3^
Monoclinic, *P*2_1_/*c*	Mo *K*α radiation, λ = 0.71073 Å
*a* = 25.1895 (16) Å	Cell parameters from 3849 reflections
*b* = 6.9411 (4) Å	θ = 1.6–28.4°
*c* = 8.9697 (6) Å	µ = 0.22 mm^−^^1^
β = 90.943 (4)°	*T* = 293 K
*V* = 1568.08 (17) Å^3^	Block, colourless
*Z* = 4	0.30 × 0.25 × 0.20 mm

### (I) 2-[(4,6-Diaminopyrimidin-2-yl)sulfanyl]-N-(naphthalen-1-yl)acetamide Data collection

**Table:**

Bruker SMART APEXII area-detector diffractometer	2095 reflections with *I* > 2σ(*I*)
ω and φ scans	*R*_int_ = 0.063
Absorption correction: multi-scan (*SADABS*; Bruker, 2008)	θ_max_ = 28.4°, θ_min_ = 1.6°
*T*_min_ = 0.752, *T*_max_ = 0.831	*h* = −33→33
14522 measured reflections	*k* = −7→9
3849 independent reflections	*l* = −11→12

### (I) 2-[(4,6-Diaminopyrimidin-2-yl)sulfanyl]-N-(naphthalen-1-yl)acetamide Refinement

**Table:**

Refinement on *F*^2^	Primary atom site location: structure-invariant direct methods
Least-squares matrix: full	Secondary atom site location: difference Fourier map
*R*[*F*^2^ > 2σ(*F*^2^)] = 0.052	Hydrogen site location: inferred from neighbouring sites
*wR*(*F*^2^) = 0.153	H-atom parameters constrained
*S* = 0.98	*w* = 1/[σ^2^(*F*_o_^2^) + (0.0671*P*)^2^ + 0.3358*P*] where *P* = (*F*_o_^2^ + 2*F*_c_^2^)/3
3849 reflections	(Δ/σ)_max_ = 0.001
208 parameters	Δρ_max_ = 0.38 e Å^−^^3^
0 restraints	Δρ_min_ = −0.23 e Å^−^^3^

### (I) 2-[(4,6-Diaminopyrimidin-2-yl)sulfanyl]-N-(naphthalen-1-yl)acetamide Special details

**Table:**

Geometry. All esds (except the esd in the dihedral angle between two l.s. planes) are estimated using the full covariance matrix. The cell esds are taken into account individually in the estimation of esds in distances, angles and torsion angles; correlations between esds in cell parameters are only used when they are defined by crystal symmetry. An approximate (isotropic) treatment of cell esds is used for estimating esds involving l.s. planes.

### (I) 2-[(4,6-Diaminopyrimidin-2-yl)sulfanyl]-N-(naphthalen-1-yl)acetamide Fractional atomic coordinates and isotropic or equivalent isotropic displacement parameters (Å^2^)

**Table:**

	*x*	*y*	*z*	*U*_iso_*/*U*_eq_	
N5	0.49526 (12)	−0.2169 (5)	0.6491 (4)	0.1242 (15)	
H5A	0.517831	−0.151383	0.599131	0.149*	
H5B	0.505205	−0.319772	0.695198	0.149*	
C1	0.15258 (14)	−0.2816 (4)	0.5842 (3)	0.0706 (9)	
H1	0.156295	−0.401205	0.538876	0.085*	
C2	0.10801 (13)	−0.2407 (5)	0.6613 (3)	0.0696 (9)	
H2	0.081326	−0.332820	0.667315	0.084*	
C3	0.10145 (10)	−0.0625 (4)	0.7317 (3)	0.0562 (7)	
C4	0.05553 (12)	−0.0167 (6)	0.8124 (3)	0.0725 (9)	
H4	0.028614	−0.107749	0.819483	0.087*	
C5	0.04960 (12)	0.1557 (6)	0.8794 (3)	0.0740 (9)	
H5	0.019065	0.181697	0.932778	0.089*	
C6	0.08929 (11)	0.2952 (5)	0.8688 (3)	0.0634 (8)	
H6	0.085036	0.413896	0.915302	0.076*	
C7	0.13418 (10)	0.2591 (4)	0.7911 (3)	0.0501 (6)	
H7	0.160100	0.354018	0.784284	0.060*	
C8	0.14195 (9)	0.0796 (4)	0.7205 (2)	0.0452 (6)	
C9	0.18804 (10)	0.0334 (4)	0.6386 (2)	0.0432 (6)	
C10	0.19288 (11)	−0.1441 (4)	0.5729 (3)	0.0576 (7)	
H10	0.223334	−0.173379	0.520215	0.069*	
C11	0.26297 (9)	0.1951 (3)	0.5182 (2)	0.0413 (6)	
C12	0.30711 (10)	0.3383 (4)	0.5478 (3)	0.0483 (6)	
H12A	0.310700	0.357511	0.654569	0.058*	
H12B	0.297111	0.460871	0.503592	0.058*	
C13	0.38351 (10)	0.0615 (3)	0.5853 (2)	0.0422 (6)	
C14	0.44443 (12)	−0.1587 (5)	0.6552 (3)	0.0688 (8)	
C15	0.40599 (12)	−0.2535 (4)	0.7352 (3)	0.0668 (8)	
H15	0.414154	−0.364339	0.789169	0.080*	
C16	0.35572 (11)	−0.1801 (4)	0.7331 (3)	0.0481 (6)	
N1	0.22916 (8)	0.1726 (3)	0.6311 (2)	0.0438 (5)	
H1A	0.232751	0.249634	0.705663	0.053*	
N2	0.34334 (7)	−0.0183 (3)	0.6559 (2)	0.0427 (5)	
N3	0.43337 (8)	0.0052 (3)	0.5783 (2)	0.0540 (6)	
N4	0.31453 (10)	−0.2587 (3)	0.8082 (2)	0.0607 (6)	
H4A	0.283743	−0.205232	0.803960	0.073*	
H4B	0.319286	−0.361782	0.859841	0.073*	
O1	0.25760 (7)	0.1132 (3)	0.39688 (16)	0.0502 (5)	
S1	0.37019 (3)	0.26816 (10)	0.47685 (8)	0.0554 (2)	

### (I) 2-[(4,6-Diaminopyrimidin-2-yl)sulfanyl]-N-(naphthalen-1-yl)acetamide Atomic displacement parameters (Å^2^)

**Table:**

	*U*^11^	*U*^22^	*U*^33^	*U*^12^	*U*^13^	*U*^23^
N5	0.0625 (18)	0.132 (3)	0.179 (3)	0.0403 (19)	0.033 (2)	0.099 (3)
C1	0.092 (2)	0.0530 (19)	0.0668 (19)	−0.0148 (18)	0.0000 (17)	−0.0022 (14)
C2	0.077 (2)	0.064 (2)	0.0671 (19)	−0.0257 (17)	−0.0050 (16)	0.0057 (15)
C3	0.0522 (17)	0.065 (2)	0.0511 (14)	−0.0107 (14)	0.0013 (12)	0.0105 (13)
C4	0.0508 (18)	0.102 (3)	0.0654 (18)	−0.0192 (18)	0.0071 (14)	0.0168 (18)
C5	0.0437 (17)	0.113 (3)	0.0657 (18)	0.0019 (19)	0.0160 (13)	0.008 (2)
C6	0.0519 (17)	0.082 (2)	0.0570 (16)	0.0086 (16)	0.0112 (13)	−0.0039 (15)
C7	0.0420 (14)	0.0617 (18)	0.0467 (14)	0.0001 (13)	0.0078 (11)	−0.0006 (12)
C8	0.0440 (14)	0.0538 (16)	0.0380 (12)	−0.0021 (12)	0.0010 (10)	0.0044 (11)
C9	0.0475 (14)	0.0455 (15)	0.0370 (11)	−0.0025 (12)	0.0051 (10)	0.0015 (10)
C10	0.0686 (19)	0.0518 (17)	0.0526 (15)	−0.0014 (15)	0.0095 (13)	−0.0034 (13)
C11	0.0460 (14)	0.0401 (14)	0.0383 (12)	0.0119 (11)	0.0109 (10)	0.0039 (10)
C12	0.0532 (16)	0.0384 (14)	0.0538 (14)	0.0032 (12)	0.0137 (12)	0.0049 (11)
C13	0.0452 (14)	0.0398 (14)	0.0417 (12)	−0.0040 (12)	0.0053 (10)	0.0008 (10)
C14	0.0571 (19)	0.070 (2)	0.080 (2)	0.0153 (16)	0.0118 (15)	0.0257 (17)
C15	0.069 (2)	0.0556 (19)	0.0763 (19)	0.0123 (16)	0.0136 (16)	0.0266 (15)
C16	0.0615 (17)	0.0387 (15)	0.0444 (13)	−0.0024 (13)	0.0094 (12)	0.0017 (11)
N1	0.0465 (12)	0.0458 (12)	0.0395 (10)	−0.0020 (10)	0.0129 (8)	−0.0056 (9)
N2	0.0477 (12)	0.0369 (12)	0.0437 (10)	−0.0037 (9)	0.0069 (9)	0.0039 (9)
N3	0.0437 (13)	0.0538 (14)	0.0646 (13)	0.0023 (11)	0.0074 (10)	0.0137 (11)
N4	0.0713 (16)	0.0460 (14)	0.0656 (14)	−0.0062 (12)	0.0217 (12)	0.0134 (11)
O1	0.0575 (11)	0.0562 (11)	0.0373 (9)	0.0086 (9)	0.0110 (7)	−0.0004 (8)
S1	0.0494 (4)	0.0524 (5)	0.0651 (4)	0.0038 (3)	0.0200 (3)	0.0208 (3)

### (I) 2-[(4,6-Diaminopyrimidin-2-yl)sulfanyl]-N-(naphthalen-1-yl)acetamide Geometric parameters (Å, º)

**Table:**

N5—C14	1.345 (4)	C9—N1	1.419 (3)
N5—H5A	0.8600	C10—H10	0.9300
N5—H5B	0.8600	C11—O1	1.234 (3)
C1—C2	1.358 (4)	C11—N1	1.343 (3)
C1—C10	1.398 (4)	C11—C12	1.512 (4)
C1—H1	0.9300	C12—S1	1.789 (2)
C2—C3	1.400 (4)	C12—H12A	0.9700
C2—H2	0.9300	C12—H12B	0.9700
C3—C4	1.411 (4)	C13—N3	1.318 (3)
C3—C8	1.424 (4)	C13—N2	1.324 (3)
C4—C5	1.349 (5)	C13—S1	1.762 (2)
C4—H4	0.9300	C14—N3	1.357 (3)
C5—C6	1.396 (4)	C14—C15	1.382 (4)
C5—H5	0.9300	C15—C16	1.365 (4)
C6—C7	1.362 (3)	C15—H15	0.9300
C6—H6	0.9300	C16—N2	1.353 (3)
C7—C8	1.413 (3)	C16—N4	1.360 (3)
C7—H7	0.9300	N1—H1A	0.8600
C8—C9	1.421 (3)	N4—H4A	0.8600
C9—C10	1.372 (3)	N4—H4B	0.8600
			
C14—N5—H5A	120.0	C1—C10—H10	119.6
C14—N5—H5B	120.0	O1—C11—N1	123.4 (2)
H5A—N5—H5B	120.0	O1—C11—C12	121.8 (2)
C2—C1—C10	120.1 (3)	N1—C11—C12	114.7 (2)
C2—C1—H1	119.9	C11—C12—S1	114.45 (17)
C10—C1—H1	119.9	C11—C12—H12A	108.6
C1—C2—C3	121.3 (3)	S1—C12—H12A	108.6
C1—C2—H2	119.4	C11—C12—H12B	108.6
C3—C2—H2	119.4	S1—C12—H12B	108.6
C2—C3—C4	122.3 (3)	H12A—C12—H12B	107.6
C2—C3—C8	119.4 (3)	N3—C13—N2	129.4 (2)
C4—C3—C8	118.3 (3)	N3—C13—S1	112.83 (17)
C5—C4—C3	121.8 (3)	N2—C13—S1	117.67 (18)
C5—C4—H4	119.1	N5—C14—N3	114.8 (3)
C3—C4—H4	119.1	N5—C14—C15	123.6 (3)
C4—C5—C6	120.0 (3)	N3—C14—C15	121.6 (3)
C4—C5—H5	120.0	C16—C15—C14	118.2 (3)
C6—C5—H5	120.0	C16—C15—H15	120.9
C7—C6—C5	120.6 (3)	C14—C15—H15	120.9
C7—C6—H6	119.7	N2—C16—N4	114.5 (2)
C5—C6—H6	119.7	N2—C16—C15	121.5 (2)
C6—C7—C8	120.9 (3)	N4—C16—C15	123.9 (2)
C6—C7—H7	119.5	C11—N1—C9	126.0 (2)
C8—C7—H7	119.5	C11—N1—H1A	117.0
C7—C8—C9	123.4 (2)	C9—N1—H1A	117.0
C7—C8—C3	118.4 (2)	C13—N2—C16	114.9 (2)
C9—C8—C3	118.2 (2)	C13—N3—C14	114.3 (2)
C10—C9—N1	121.4 (2)	C16—N4—H4A	120.0
C10—C9—C8	120.2 (2)	C16—N4—H4B	120.0
N1—C9—C8	118.3 (2)	H4A—N4—H4B	120.0
C9—C10—C1	120.8 (3)	C13—S1—C12	100.80 (11)
C9—C10—H10	119.6		
			
C10—C1—C2—C3	−0.5 (5)	O1—C11—C12—S1	−42.3 (3)
C1—C2—C3—C4	180.0 (3)	N1—C11—C12—S1	140.88 (18)
C1—C2—C3—C8	1.0 (4)	N5—C14—C15—C16	179.9 (3)
C2—C3—C4—C5	−179.8 (3)	N3—C14—C15—C16	1.3 (5)
C8—C3—C4—C5	−0.8 (4)	C14—C15—C16—N2	−0.4 (4)
C3—C4—C5—C6	0.7 (5)	C14—C15—C16—N4	−179.3 (3)
C4—C5—C6—C7	0.0 (5)	O1—C11—N1—C9	10.4 (4)
C5—C6—C7—C8	−0.6 (4)	C12—C11—N1—C9	−172.8 (2)
C6—C7—C8—C9	−179.7 (2)	C10—C9—N1—C11	31.7 (4)
C6—C7—C8—C3	0.5 (4)	C8—C9—N1—C11	−150.3 (2)
C2—C3—C8—C7	179.3 (2)	N3—C13—N2—C16	0.8 (4)
C4—C3—C8—C7	0.2 (4)	S1—C13—N2—C16	177.84 (17)
C2—C3—C8—C9	−0.6 (4)	N4—C16—N2—C13	178.5 (2)
C4—C3—C8—C9	−179.7 (2)	C15—C16—N2—C13	−0.5 (3)
C7—C8—C9—C10	−180.0 (2)	N2—C13—N3—C14	0.0 (4)
C3—C8—C9—C10	−0.1 (3)	S1—C13—N3—C14	−177.2 (2)
C7—C8—C9—N1	2.1 (3)	N5—C14—N3—C13	−179.8 (3)
C3—C8—C9—N1	−178.1 (2)	C15—C14—N3—C13	−1.1 (4)
N1—C9—C10—C1	178.4 (2)	N3—C13—S1—C12	−165.84 (19)
C8—C9—C10—C1	0.6 (4)	N2—C13—S1—C12	16.6 (2)
C2—C1—C10—C9	−0.2 (4)	C11—C12—S1—C13	−64.63 (19)

### (I) 2-[(4,6-Diaminopyrimidin-2-yl)sulfanyl]-N-(naphthalen-1-yl)acetamide Hydrogen-bond geometry (Å, º)

**Table:**

*D*—H···*A*	*D*—H	H···*A*	*D*···*A*	*D*—H···*A*
N5—H5*A*···N3^i^	0.86	2.27	3.110 (4)	167
N1—H1*A*···O1^ii^	0.86	2.05	2.890 (3)	165
N4—H4*B*···O1^iii^	0.86	2.36	2.964 (3)	127
C12—H12*A*···O1^ii^	0.97	2.58	3.408 (3)	143

Symmetry codes: (i) −*x*+1, −*y*, −*z*+1; (ii) *x*, −*y*+1/2, *z*+1/2; (iii) *x*, −*y*−1/2, *z*+1/2.

### (II) 2-[(4,6-Diaminopyrimidin-2-yl)sulfanyl]-N-(4-fluorophenyl)acetamide Crystal data

**Table:**

C_12_H_12_FN_5_OS	*F*(000) = 608
*M**_r_* = 293.33	*D*_x_ = 1.441 Mg m^−^^3^
Monoclinic, *P*2_1_/*c*	Mo *K*α radiation, λ = 0.71073 Å
*a* = 21.7358 (7) Å	Cell parameters from 3312 reflections
*b* = 7.3726 (3) Å	θ = 1.9–28.3°
*c* = 8.4487 (3) Å	µ = 0.25 mm^−^^1^
β = 93.092 (1)°	*T* = 293 K
*V* = 1351.93 (9) Å^3^	Block, colourless
*Z* = 4	0.31 × 0.25 × 0.20 mm

### (II) 2-[(4,6-Diaminopyrimidin-2-yl)sulfanyl]-N-(4-fluorophenyl)acetamide Data collection

**Table:**

Bruker SMART APEXII area-detector diffractometer	2829 reflections with *I* > 2σ(*I*)
ω and φ scans	*R*_int_ = 0.025
Absorption correction: multi-scan (*SADABS*; Bruker, 2008)	θ_max_ = 28.3°, θ_min_ = 1.9°
*T*_min_ = 0.652, *T*_max_ = 0.753	*h* = −28→28
12316 measured reflections	*k* = −5→9
3312 independent reflections	*l* = −7→11

### (II) 2-[(4,6-Diaminopyrimidin-2-yl)sulfanyl]-N-(4-fluorophenyl)acetamide Refinement

**Table:**

Refinement on *F*^2^	Primary atom site location: structure-invariant direct methods
Least-squares matrix: full	Secondary atom site location: difference Fourier map
*R*[*F*^2^ > 2σ(*F*^2^)] = 0.037	Hydrogen site location: inferred from neighbouring sites
*wR*(*F*^2^) = 0.109	H-atom parameters constrained
*S* = 1.05	*w* = 1/[σ^2^(*F*_o_^2^) + (0.055*P*)^2^ + 0.3099*P*] where *P* = (*F*_o_^2^ + 2*F*_c_^2^)/3
3312 reflections	(Δ/σ)_max_ = 0.001
181 parameters	Δρ_max_ = 0.22 e Å^−^^3^
0 restraints	Δρ_min_ = −0.22 e Å^−^^3^

### (II) 2-[(4,6-Diaminopyrimidin-2-yl)sulfanyl]-N-(4-fluorophenyl)acetamide Special details

**Table:**

Geometry. All esds (except the esd in the dihedral angle between two l.s. planes) are estimated using the full covariance matrix. The cell esds are taken into account individually in the estimation of esds in distances, angles and torsion angles; correlations between esds in cell parameters are only used when they are defined by crystal symmetry. An approximate (isotropic) treatment of cell esds is used for estimating esds involving l.s. planes.

### (II) 2-[(4,6-Diaminopyrimidin-2-yl)sulfanyl]-N-(4-fluorophenyl)acetamide Fractional atomic coordinates and isotropic or equivalent isotropic displacement parameters (Å^2^)

**Table:**

	*x*	*y*	*z*	*U*_iso_*/*U*_eq_	
C1	0.43036 (9)	−0.1374 (4)	0.4152 (3)	0.0777 (6)	
C2	0.42379 (9)	0.0399 (4)	0.3738 (3)	0.0824 (6)	
H2	0.450358	0.092466	0.304272	0.099*	
C3	0.37714 (8)	0.1422 (3)	0.4359 (2)	0.0658 (4)	
H3	0.372002	0.263568	0.408377	0.079*	
C4	0.33831 (6)	0.0600 (2)	0.53966 (17)	0.0480 (3)	
C5	0.34659 (8)	−0.1211 (2)	0.5791 (2)	0.0605 (4)	
H5	0.320667	−0.175666	0.649109	0.073*	
C6	0.39282 (10)	−0.2211 (3)	0.5158 (3)	0.0746 (5)	
H6	0.398185	−0.342957	0.541273	0.089*	
C7	0.26854 (6)	0.32063 (19)	0.58470 (16)	0.0432 (3)	
C8	0.21206 (7)	0.36589 (19)	0.67409 (17)	0.0482 (3)	
H8A	0.210250	0.496199	0.688963	0.058*	
H8B	0.216105	0.310216	0.778152	0.058*	
C9	0.13013 (6)	0.07360 (17)	0.65615 (14)	0.0378 (3)	
C10	0.06207 (7)	−0.1564 (2)	0.67975 (18)	0.0501 (3)	
C11	0.10494 (7)	−0.2466 (2)	0.77884 (18)	0.0505 (3)	
H11	0.095821	−0.358065	0.823502	0.061*	
C12	0.16170 (7)	−0.16497 (18)	0.80890 (15)	0.0415 (3)	
F1	0.47632 (7)	−0.2347 (3)	0.3527 (2)	0.1187 (6)	
N1	0.28896 (5)	0.15094 (17)	0.60807 (15)	0.0480 (3)	
H1	0.269193	0.087989	0.674351	0.058*	
N2	0.07407 (5)	0.00964 (16)	0.61864 (14)	0.0452 (3)	
N3	0.17559 (5)	−0.00336 (14)	0.74173 (12)	0.0390 (2)	
N4	0.20765 (7)	−0.23810 (17)	0.90067 (16)	0.0546 (3)	
H4A	0.242409	−0.182765	0.912926	0.066*	
H4B	0.202291	−0.340228	0.947136	0.066*	
N5	0.00588 (8)	−0.2236 (2)	0.6394 (2)	0.0815 (5)	
H5A	−0.019470	−0.162080	0.579047	0.098*	
H5B	−0.004701	−0.328149	0.674068	0.098*	
O1	0.29125 (5)	0.43187 (15)	0.49776 (14)	0.0597 (3)	
S1	0.14082 (2)	0.29101 (5)	0.57535 (5)	0.05097 (13)	

### (II) 2-[(4,6-Diaminopyrimidin-2-yl)sulfanyl]-N-(4-fluorophenyl)acetamide Atomic displacement parameters (Å^2^)

**Table:**

	*U*^11^	*U*^22^	*U*^33^	*U*^12^	*U*^13^	*U*^23^
C1	0.0526 (10)	0.0988 (16)	0.0819 (13)	0.0139 (10)	0.0035 (9)	−0.0235 (12)
C2	0.0581 (11)	0.1083 (18)	0.0830 (13)	−0.0068 (11)	0.0248 (10)	−0.0037 (13)
C3	0.0548 (9)	0.0710 (11)	0.0728 (11)	−0.0055 (8)	0.0142 (8)	0.0061 (9)
C4	0.0429 (7)	0.0532 (8)	0.0478 (7)	−0.0038 (6)	−0.0002 (6)	−0.0007 (6)
C5	0.0577 (9)	0.0563 (10)	0.0677 (10)	0.0023 (7)	0.0049 (7)	0.0015 (8)
C6	0.0682 (11)	0.0665 (12)	0.0885 (14)	0.0138 (9)	−0.0011 (10)	−0.0103 (10)
C7	0.0453 (7)	0.0420 (7)	0.0418 (6)	−0.0102 (5)	−0.0029 (5)	0.0014 (5)
C8	0.0597 (8)	0.0347 (7)	0.0508 (7)	−0.0064 (6)	0.0081 (6)	−0.0023 (6)
C9	0.0447 (6)	0.0333 (6)	0.0366 (6)	0.0004 (5)	0.0123 (5)	−0.0009 (5)
C10	0.0498 (8)	0.0469 (8)	0.0543 (8)	−0.0080 (6)	0.0102 (6)	0.0061 (6)
C11	0.0604 (9)	0.0395 (7)	0.0521 (8)	−0.0086 (6)	0.0088 (7)	0.0089 (6)
C12	0.0551 (7)	0.0340 (6)	0.0361 (6)	0.0012 (5)	0.0082 (5)	−0.0014 (5)
F1	0.0774 (9)	0.1484 (14)	0.1323 (13)	0.0357 (9)	0.0230 (8)	−0.0415 (11)
N1	0.0499 (6)	0.0442 (6)	0.0508 (6)	−0.0030 (5)	0.0106 (5)	0.0070 (5)
N2	0.0442 (6)	0.0422 (6)	0.0499 (6)	−0.0029 (5)	0.0079 (5)	0.0056 (5)
N3	0.0467 (6)	0.0320 (5)	0.0390 (5)	−0.0001 (4)	0.0081 (4)	0.0003 (4)
N4	0.0677 (8)	0.0397 (6)	0.0557 (7)	−0.0015 (6)	−0.0045 (6)	0.0089 (5)
N5	0.0586 (9)	0.0747 (11)	0.1098 (14)	−0.0267 (8)	−0.0091 (8)	0.0363 (10)
O1	0.0564 (6)	0.0514 (6)	0.0719 (7)	−0.0071 (5)	0.0085 (5)	0.0188 (5)
S1	0.0472 (2)	0.0404 (2)	0.0655 (2)	−0.00120 (14)	0.00437 (16)	0.01690 (16)

### (II) 2-[(4,6-Diaminopyrimidin-2-yl)sulfanyl]-N-(4-fluorophenyl)acetamide Geometric parameters (Å, º)

**Table:**

C1—C6	1.358 (3)	C8—H8B	0.9700
C1—C2	1.359 (3)	C9—N3	1.3207 (17)
C1—F1	1.359 (2)	C9—N2	1.3288 (17)
C2—C3	1.389 (3)	C9—S1	1.7625 (13)
C2—H2	0.9300	C10—N5	1.345 (2)
C3—C4	1.388 (2)	C10—N2	1.3594 (18)
C3—H3	0.9300	C10—C11	1.388 (2)
C4—C5	1.386 (2)	C11—C12	1.384 (2)
C4—N1	1.4144 (19)	C11—H11	0.9300
C5—C6	1.378 (3)	C12—N4	1.3438 (19)
C5—H5	0.9300	C12—N3	1.3606 (17)
C6—H6	0.9300	N1—H1	0.8600
C7—O1	1.2227 (16)	N4—H4A	0.8600
C7—N1	1.3386 (19)	N4—H4B	0.8600
C7—C8	1.513 (2)	N5—H5A	0.8600
C8—S1	1.8054 (15)	N5—H5B	0.8600
C8—H8A	0.9700		
			
C6—C1—C2	122.69 (18)	H8A—C8—H8B	107.7
C6—C1—F1	118.8 (2)	N3—C9—N2	128.87 (12)
C2—C1—F1	118.5 (2)	N3—C9—S1	119.50 (10)
C1—C2—C3	119.57 (19)	N2—C9—S1	111.64 (10)
C1—C2—H2	120.2	N5—C10—N2	115.21 (15)
C3—C2—H2	120.2	N5—C10—C11	123.17 (14)
C4—C3—C2	118.9 (2)	N2—C10—C11	121.61 (14)
C4—C3—H3	120.6	C12—C11—C10	117.71 (13)
C2—C3—H3	120.6	C12—C11—H11	121.1
C5—C4—C3	119.78 (16)	C10—C11—H11	121.1
C5—C4—N1	116.72 (14)	N4—C12—N3	114.66 (13)
C3—C4—N1	123.49 (16)	N4—C12—C11	123.98 (13)
C6—C5—C4	120.67 (18)	N3—C12—C11	121.34 (13)
C6—C5—H5	119.7	C7—N1—C4	129.45 (13)
C4—C5—H5	119.7	C7—N1—H1	115.3
C1—C6—C5	118.4 (2)	C4—N1—H1	115.3
C1—C6—H6	120.8	C9—N2—C10	114.92 (12)
C5—C6—H6	120.8	C9—N3—C12	115.35 (11)
O1—C7—N1	125.03 (14)	C12—N4—H4A	120.0
O1—C7—C8	121.11 (13)	C12—N4—H4B	120.0
N1—C7—C8	113.84 (12)	H4A—N4—H4B	120.0
C7—C8—S1	113.63 (10)	C10—N5—H5A	120.0
C7—C8—H8A	108.8	C10—N5—H5B	120.0
S1—C8—H8A	108.8	H5A—N5—H5B	120.0
C7—C8—H8B	108.8	C9—S1—C8	103.11 (7)
S1—C8—H8B	108.8		
			
C6—C1—C2—C3	0.2 (3)	O1—C7—N1—C4	−1.5 (2)
F1—C1—C2—C3	179.92 (18)	C8—C7—N1—C4	177.03 (13)
C1—C2—C3—C4	0.2 (3)	C5—C4—N1—C7	−176.10 (15)
C2—C3—C4—C5	−0.1 (3)	C3—C4—N1—C7	3.0 (2)
C2—C3—C4—N1	−179.19 (16)	N3—C9—N2—C10	1.3 (2)
C3—C4—C5—C6	−0.3 (3)	S1—C9—N2—C10	−178.73 (10)
N1—C4—C5—C6	178.83 (15)	N5—C10—N2—C9	−178.64 (15)
C2—C1—C6—C5	−0.6 (3)	C11—C10—N2—C9	2.5 (2)
F1—C1—C6—C5	179.66 (18)	N2—C9—N3—C12	−4.68 (19)
C4—C5—C6—C1	0.7 (3)	S1—C9—N3—C12	175.34 (9)
O1—C7—C8—S1	96.18 (14)	N4—C12—N3—C9	−177.50 (12)
N1—C7—C8—S1	−82.44 (14)	C11—C12—N3—C9	4.35 (18)
N5—C10—C11—C12	178.71 (16)	N3—C9—S1—C8	−11.34 (11)
N2—C10—C11—C12	−2.5 (2)	N2—C9—S1—C8	168.68 (10)
C10—C11—C12—N4	−179.08 (14)	C7—C8—S1—C9	91.88 (11)
C10—C11—C12—N3	−1.1 (2)		

### (II) 2-[(4,6-Diaminopyrimidin-2-yl)sulfanyl]-N-(4-fluorophenyl)acetamide Hydrogen-bond geometry (Å, º)

**Table:**

*D*—H···*A*	*D*—H	H···*A*	*D*···*A*	*D*—H···*A*
N1—H1···N3	0.86	2.25	2.990 (2)	145
C3—H3···O1	0.93	2.31	2.903 (2)	121
N5—H5*A*···N2^i^	0.86	2.29	3.139 (2)	169
N4—H4*A*···O1^ii^	0.86	2.23	2.9852 (18)	146
C2—H2···F1^iii^	0.93	2.48	3.404 (3)	172

Symmetry codes: (i) −*x*, −*y*, −*z*+1; (ii) *x*, −*y*+1/2, *z*+1/2; (iii) −*x*+1, *y*+1/2, −*z*+1/2.
